# Do repeat 6-minute walk tests provide clinically meaningful information in symptomatic heart failure?

**DOI:** 10.1093/eschf/xvag209

**Published:** 2026-07-30

**Authors:** Trond J Cooper, Pierpaolo Pellicori, Hanne Brit Hetland, Kenneth Dickstein

**Affiliations:** Department of Clinical Science, University of Bergen, Bergen, Vestland, Norway; Department of Cardiology, Leif Larsens gate 8, Stavanger, Rogaland, Norway; School of Cardiovascular and Metabolic Health, University of Glasgow Institute of Cardiovascular and Medical Sciences, University of Glasgow, Glasgow, UK; Department of Biostatistics, Stavanger University Hospital, Stavanger 4068, Norway; Department of Clinical Science, University of Bergen, Bergen, Vestland, Norway; Department of Cardiology, Leif Larsens gate 8, Stavanger, Rogaland, Norway

**Keywords:** Exercise performance, Heart failure, Six-minute walk test

## Abstract

**Background and Aims:**

Two baseline 6-minute walk tests (6MWTs) have been recommended to mitigate a potential learning effect when assessing exercise performance, but the magnitude of any learning effect in heart failure with reduced ejection fraction (HFrEF) is uncertain. Using the Sildenafil in Heart Failure (SilHF) trial, in which two baseline 6MWTs were performed (screening and inclusion), we assessed a potential learning effect in this population. We also assessed whether choice of baseline test affected associations with longitudinal patient-reported outcomes and compared our findings to reports in the current literature.

**Methods:**

In SilHF, 69 patients with HFrEF and evidence of pulmonary hypertension performed 6MWTs at screening and again at study inclusion. Tests were performed no more than 2 weeks apart on unchanged optimal medical therapy. Only the inclusion 6MWT was used for efficacy evaluation of sildenafil. Screening and inclusion distances were compared using a paired *t*-test. Additionally, we assessed whether each baseline 6MWT (screening and inclusion) correlated with longitudinal patient-reported outcome measures. Outcomes included the Kansas City Cardiomyopathy Questionnaire and the EuroQoL-5D visual analogue scale, assessed at 24 weeks.

**Results:**

Although the inclusion 6MWT distance was statistically significantly higher than the screening test (+11.3 m, 95% confidence interval 2.6–20.1, *P* = .012), the two baseline tests demonstrated very similar correlation coefficients with longitudinal patient-reported outcomes.

**Conclusion:**

We observed a small increase in 6MWD between repeat baseline tests, consistent with a modest learning effect that is unlikely to be clinically meaningful. Both initial 6MWTs showed similar significant correlations with other longitudinal patient-reported outcome measures. In symptomatic HFrEF with pulmonary hypertension, a single baseline 6MWT may be sufficient to assess functional capacity.

## Graphical Abstract

**Graphical Abstract:**
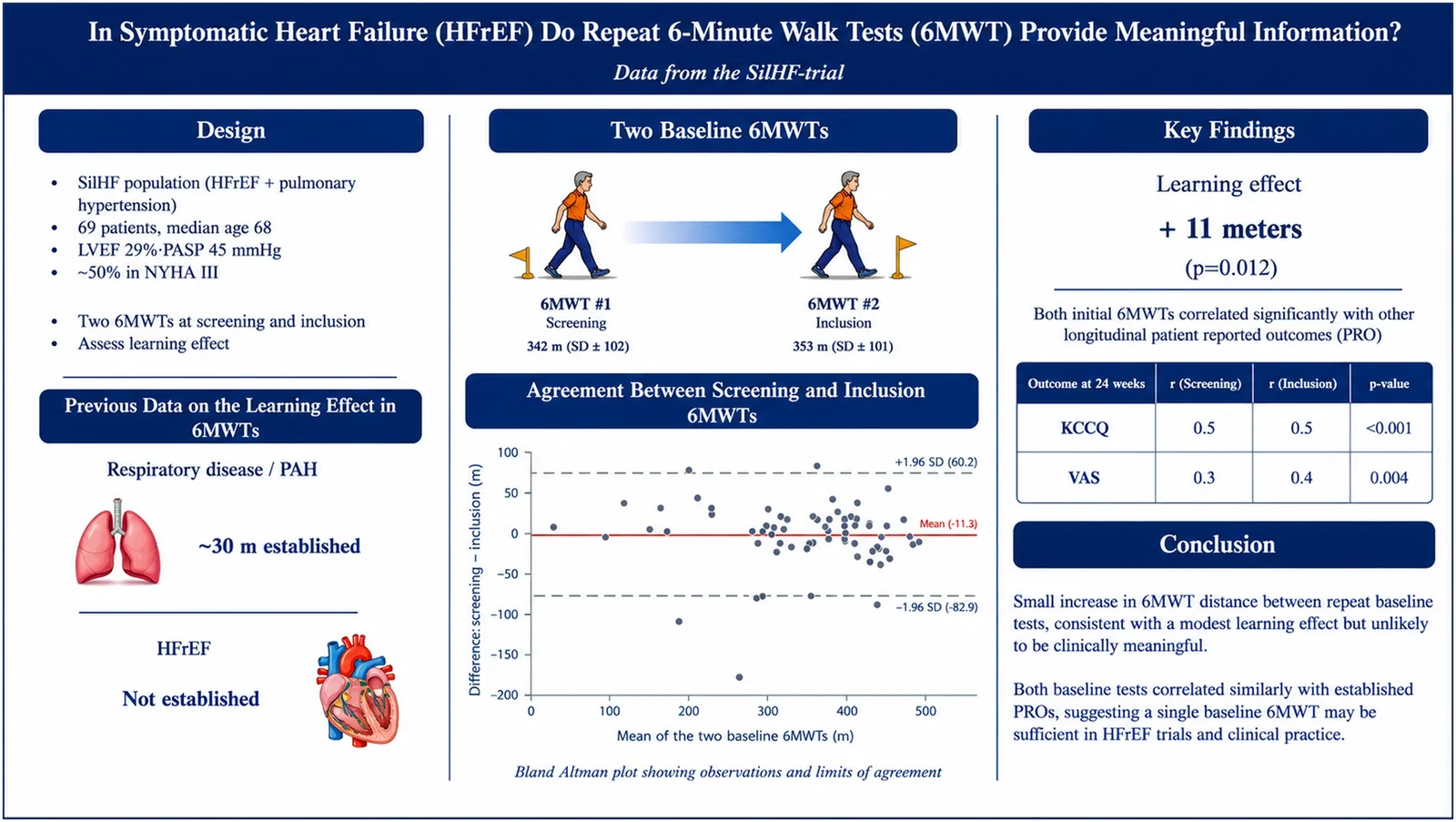

## Introduction

The 6-minute walk test (6MWT) is a validated and widely used measure of submaximal exercise capacity. Initially used in patients with chronic respiratory disease,^1,2^ the 6MWT has become one of the most frequently used outcome measures in heart failure (HF) trials, particularly in smaller studies where symptom-related endpoints are important. Several factors influence 6MWT performance, including baseline functional status, comorbidities, and demographic and anthropometric characteristics.^3^ There are also protocol-related factors such as encouragement and familiarization with the test. A potential limitation of the 6MWT is the learning effect where repeat testing improves performance independent of a true physiological change. This effect is well documented in patients with chronic respiratory disease^4–6^ and repeat baseline tests have been advocated in clinical practice guidelines.^7^

A learning effect has also been reported in other groups such as patients with pulmonary arterial hypertension (PAH) (PH group 1),^8^ long COVID as well as in healthy volunteers^9^ but, in patients with symptomatic HF, small studies have shown varying results.^10–12^ To our knowledge, no major cardiology society routinely recommends repeat baseline 6MWT testing, but this has been advocated in prior HF literature.^3^

Using data from the Sildenafil in Heart Failure (SilHF) trial, we assessed repeat baseline 6MWT performance in symptomatic patients with HF with reduced ejection fraction (HFrEF) and pulmonary hypertension (PH) and interpreted our findings in the context of the existing literature in HF, chronic respiratory disease, and PAH. Clarifying the magnitude of the learning effect in HFrEF may inform trial design and the interpretation of the 6MWT in clinical practice.

## Methods

This analysis used the data from the SilHF trial, previously published.^13^ Briefly, SilHF was a randomized, double-blind, placebo-controlled trial evaluating phosphodiesterase 5 inhibition in patients with HFrEF and echocardiographic evidence of PH due to left heart disease (group 2 PH). Participants were assigned to sildenafil or placebo in a 2:1 ratio for 24 weeks. The primary endpoints were patient global assessment and the 6MWT. Secondary endpoints included changes in quality of life (QoL) assessed using the EuroQoL-5D (EQ-5D) and Kansas City Cardiomyopathy Questionnaires (KCCQ). Patient-reported outcomes have previously been shown to provide sensitive markers of clinical outcomes in patients with HF.^14^ The trial was neutral.

Two 6MWTs were performed by trained research nurses at screening and at inclusion, no more than 2 weeks apart and usually only a few days apart. Per protocol, eligible patients were required to be on stable optimal guideline-directed therapy with unchanged doses for at least 30 days prior to inclusion. Background therapy therefore remained unchanged during the repeat 6MWT. Both tests were also performed before any initiation or up-titration of study drug. In the main study, only the second inclusion test was used for efficacy evaluation.

### Statistical analysis

Continuous variables are summarized as mean (standard deviation, *SD*) or median (interquartile range, IQR) and categorical variables as counts (percentages). The differences between screening and inclusion 6MWT distance were assessed using a two-sided paired *t*-test (at 0.05 level). The distribution of the paired differences was inspected for approximate normality before test application. One patient was excluded owing to an implausible 219 m difference between the screening and inclusion 6MWT distances, consistent with a recording error; the paired comparison therefore included 69 patients. Agreement between the two baseline tests was assessed using Bland–Altman analysis, reporting the mean difference and 95% limits of agreement (mean ± 1.96 *SD* of the differences). To explore whether the choice of baseline 6MWT (screening and inclusion) influenced associations with longitudinal patient-reported outcomes, the correlations of each baseline 6MWT with the EQ-5D VAS and KCCQ scores at 24 weeks were assessed using Spearman rank correlation coefficients. Analyses were performed using R version 4.3.2 (R Foundation for Statistical Computing, Vienna, Austria) in RStudio version 2023.12.0.

### Outcome measures

The 6MWT was performed based on the protocol described by Guyatt et al.^15^ Results were recorded on a standardized sheet according to protocol and subsequently entered into an electronic case report form.

The EQ-5D is a standardized non–disease-specific instrument measuring health outcome.^16^ It has been widely used in clinical trials for assessing QoL and is a valid QoL assessment including HF populations.^17^ The EQ-5D VAS is on a vertical, graduated (0–100 points) scale, with a score of 100 representing ‘best imaginable health state’ and 0 representing ‘worst imaginable health state’.

The KCCQ is a self-administered tool for measuring health-related QoL in HF.^18^ It is comprised of a 23-item questionnaire that quantifies functional capacity, symptoms (frequency, severity, and recent change), social function, self-efficacy and knowledge, and QoL. The overall summary score is a weighted average of the different domains and has a continuous scale that ranges from 0 to 100, with a higher score indicating a better QoL.

## Results

A total of 69 patients were included. The median age was 68 years [IQR 62–74], 16% were women, and 46% had atrial fibrillation. The median left ventricular ejection fraction was 29% [IQR 24–35], and the median estimated pulmonary artery systolic pressure was 45 mmHg [IQR 42–55]. Forty-six percent of patients were in NYHA class II and 54% in class III. The median baseline KCCQ clinical summary score was 72. All 69 patients completed two baseline 6MWTs. Baseline characteristics of the study population are presented in *Table 1*.

**Table 1:** Baseline characteristics of patients included in SilHF (*n* = 69)

Age, years (IQR)	68 (62–74)
Female (%)	11 (16)
Weight, kg (IQR)	81 (70–88)
Atrial fibrillation n (%)	32 (46)
LVEF, % (IQR)	29 (24–35)
NYHA class II n (%)	32 (46)
NYHA class III n (%)	37 (54)
PASP, mmHg (IQR)	45 (42–55)
6MWT, m (IQR)	374 (312–427)
KCCQ clinical score (IQR)	72 (58–83)

LVEF, left ventricular ejection fraction; PASP, pulmonary artery systolic pressure; 6MWT, 6-min walk test distance; KCCQ, Kansas City Cardiomyopathy Questionnaire; IQR, interquartile range

The mean distance walked increased from 341.5 m (*SD* 101.9) at screening to 352.8 m (*SD* 101.0) at inclusion, corresponding to a mean difference of 11.3 m (95% confidence interval: 2.6–20.1) (*P* = .012). On Bland–Altman analysis (*Figure 1*; differences plotted as screening minus inclusion), the limits of agreement were −82.9 to +60.2 m and four observations lay outside these limits (±1.96 *SD*). Correlations between the two baseline 6MWTs and the 24-week patient-reported outcomes were similar (*Table 2*): for the KCCQ *r* = 0.5 for both screening and inclusion (both *P* < .001); for the EQ-5D VAS r = 0.3 (screening *P* = .009) and *r* = 0.4 (inclusion *P* = .004). Both baseline tests also correlated similarly with the 24-week 6MWT: *r* = 0.7 and *r* = 0.8 for screening and inclusion, respectively.

**Figure 1:**
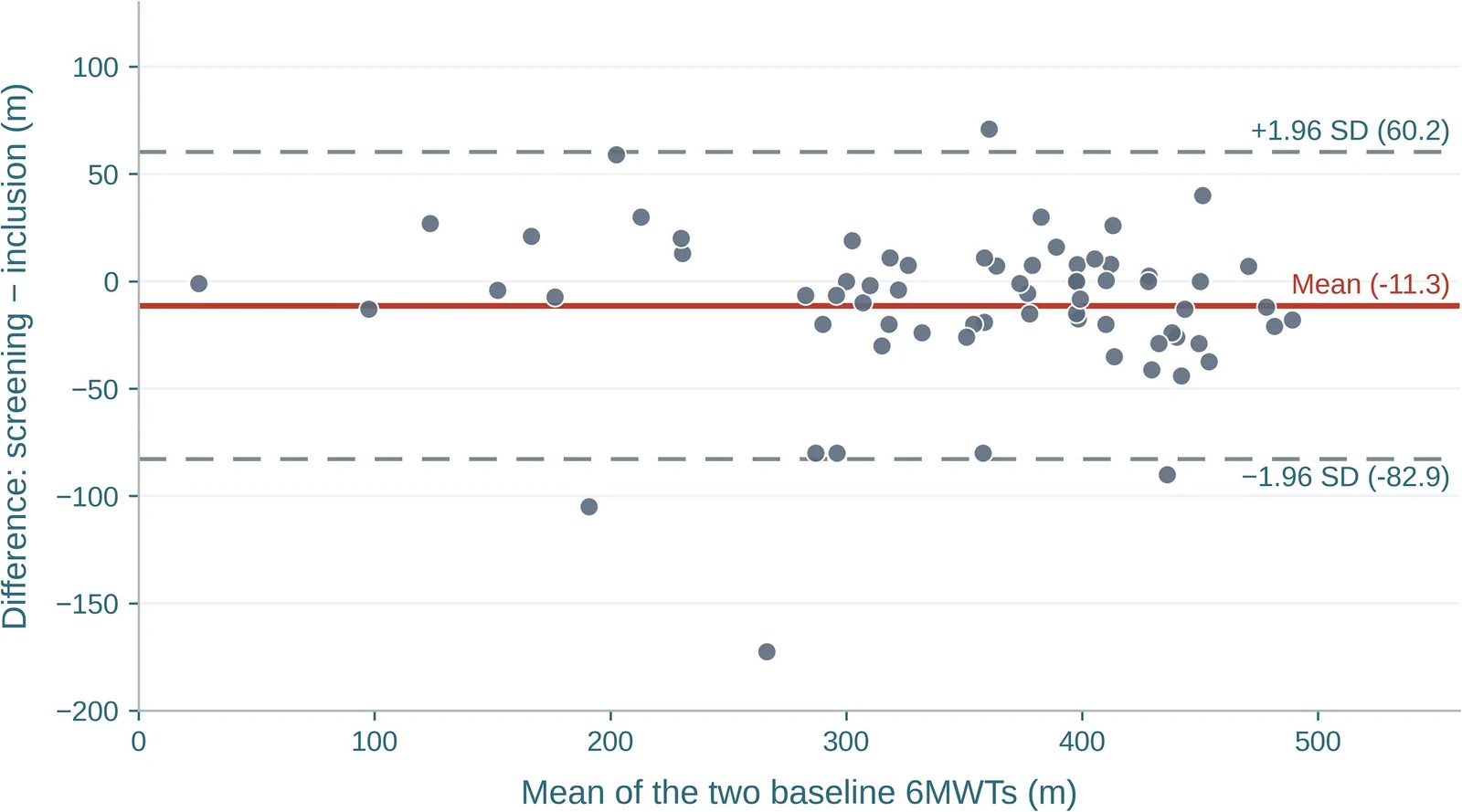
Bland–Altman plot showing agreement between the two baseline 6MWT measurements. The red line represents the mean difference and the grey dashed lines represent the limits of agreement (±1.96 SD). A negative value indicates a higher 6MWT distance at inclusion compared to screening. 6MWT, 6-minute walk test; SD, standard deviation.

**Table 2:** Correlations between baseline 6MWTs and 24-week patient reported outcomes

Outcome (24 weeks)	r (Screening 6MWT)	*P*-value	r (Inclusion 6MWT)	*P*-value
KCCQ	0.5	<.001	0.5	<.001
VAS	0.3	.009	0.4	.004
6MWT (24 weeks)	0.7	<.001	0.8	<.001

6MWT, 6-minute walk test; KCCQ, Kansas City Cardiomyopathy Questionnaire; VAS, Visual Analogue Scale

## Discussion

In this analysis of repeat baseline 6MWTs in symptomatic HFrEF patients enrolled in the SilHF trial, we observed a small but statistically significant increase in distance walked between screening and inclusion testing (*P* = .012). However, the absolute difference (11.3 m) is unlikely to be clinically meaningful, as published estimates of the minimal clinically important difference for 6MWD in chronic HF are typically in the range of 25–32 m.^19,20^

As an exploratory analysis, we examined whether use of either baseline 6MWT (screening vs inclusion) influenced associations between baseline functional capacity and subsequent patient-reported outcomes. Importantly, correlations between each baseline 6MWT and longitudinal patient-reported outcome measures (EQ-5D VAS and KCCQ) were similar. While these correlations do not assess the learning effect directly, they provide a pragmatic indication of whether the modest baseline improvement observed in our trial affected downstream interpretation of functional status. Collectively, these findings suggest that repeating baseline 6MWT in symptomatic HFrEF does not substantially alter the relationship between baseline functional capacity and subsequent health status. As in previous studies, we found moderate correlations between 6MWT distance and validated measures of health status including the KCCQ and EQ-5D.^14^

Because the SilHF trial was neutral relative to placebo, it offered a useful setting in which to examine the stability of baseline functional assessment, although we cannot exclude temporal changes in symptoms or performance status over follow-up. Consistent with previous evidence, we observed moderate correlations between 6MWT distance and EQ-5D VAS and KCCQ. Notably, these associations were modest and the 6MWT shared only about a quarter of its variance with the KCCQ (*r* approximately 0.5, *r*-squared approximately 0.25) and less with the EQ-5D VAS (*r* approximately 0.3–0.4). This indicates that the 6MWT and patient-reported instruments capture related but distinct dimensions, namely objective functional capacity versus perceived health status, and are complementary. Both baseline 6MWTs correlated with the 24-week 6MWT (*r* = 0.7 and 0.8) as expected for repeated measurements of the same test, and this correlation is likely to be independent of any potential learning effect.

### Comparison with previous studies

Previous studies assessing the learning effect of repeat 6MWTs in patients with HF have reported heterogeneous results. Lans and co-workers reported minimal learning effects during repeated serial testing over one year.^21^ In contrast, Adsett and colleagues studied 88 patients undergoing two tests within 90 min and found a statistically significant increase of 12 m, with the greatest improvement among those with a higher baseline walking distance, suggesting that disease severity may have attenuated the learning effect.^22^ Similarly, Uszko-Lencer et al. reported that the likelihood of a meaningful increase on the second 6MWT decreased with increasing symptom severity.

Our findings are consistent with those of Adsett and colleagues, with a similar increase between the first and second 6MWT distance (11.3 vs 12 m). Nevertheless, the observed increase in HFrEF appears smaller than that reported in other patient populations where learning effects are more pronounced, such as chronic obstructive pulmonary disease (COPD) and pulmonary arterial hypertension (PH group 1).

### Why learning effects may differ in HFrEF vs respiratory disease and PAH

The learning effect in 6MWT performance has been well documented in chronic respiratory disease, including large retrospective analyses. Jenkins and colleagues reported that the majority of patients with chronic lung disease (including COPD, interstitial lung disease, asthma, and bronchiectasis) increased walking distance on the second test performed on the same day, with a mean increase of 30 m.^5^ Hernandes et al. similarly reported a mean increase of 27 m in a large COPD cohort (*n* = 1514) between two tests done on separate days.^4^ These findings suggest that the methodological value of routine repeat baseline 6MWT testing in symptomatic HFrEF may be limited, in contrast to recommendations commonly adopted in chronic respiratory disease.

Several mechanisms may explain why the learning effect appears more limited in symptomatic HFrEF. Anxiety and fear of exertional dyspnoea may influence pacing and performance in respiratory disease. In COPD^23,24^ and PAH,^25^ this is well described and may contribute to larger improvements on repeat testing. In contrast, Uszko-Lencer^12^ and colleagues did not identify anxiety as a major contributor to learning effect in HF. It is plausible that patients may be more ‘conditioned’ to exertional symptoms, resulting in less behavioural adaptation between repeated tests. Moreover, in patients with HF, exercise capacity is primarily driven by cardiac impairment and peripheral skeletal muscle dysfunction, leading patients to reach a physiological ceiling during exertion.

This is supported by the observation that learning effects diminish in those with lower baseline 6MWT distance and disease severity^8^; however, all these suggested mechanisms remain speculative. A summary of key studies across major relevant populations is provided in *Table 3*.

**Table 3:** Studies evaluating the learning effect in the 6-minute walk test (6MWT) across different patient populations

First author	Journal	N	Population	Protocol	Mean Change (2nd vs 1st test)	Notes
Heart Failure (HFrEF)						
SilHF (current)	ESC HF	69	HFrEF + PH	Two tests ∼1 week	+11.3 m (*P* .012)	Small learning effect, similar correlations with LPRO
Adsett (2011)^22^	Eur J Cardiovasc Prev Rehabil	88	CHF	Two tests on same day	+12 m (*P* < .05)	More pronounced effect in patients with higher baseline distance
Uszko-Lencer (2017)^12^	Int J Cardiol	337	CHF	Two tests on consecutive days	+31 m	Smaller effect with advanced age, higher NYHA class and impaired respiratory function
Lans (2020)^21^	Clin Physiol Funct Imaging	46	CHF	Repeat baseline testing	+9 m	Change considered below clinical relevance
Chronic Respiratory Disease						
Hernandes (2011)^4^	Eur Respir J	1514	COPD	Subsequent days	+27 m	Supports guideline recommendations for duplicate baseline testing
Jenkins (2010)^5^	Respirology	349	COPD/ILD/asthma/bronchiectasis	Two tests on same day	+30 m	Learning effect consistent across respiratory diagnoses
Sciurba (2003)	Am J Respir Crit Care Med	761	COPD	Subsequent days	+22 m	Significant learning effect but wide variability
Pulmonary Arterial Hypertension						
Spencer (2018)^8^	Chronic Respiratory Disease	214	PAH	Repeat baseline testing	+19 m	Less pronounced learning effect in more advanced disease

Abbreviations: 6MWD, 6-minute walk distance; CHF, chronic heart failure; COPD, chronic obstructive pulmonary disease; DLCO, diffusing capacity; ILD, interstitial lung disease, PH, pulmonary hypertension, LPRO; longitudinal patient-reported outcomes

### Limitations

Several limitations should be acknowledged. First, SilHF was designed and conducted as a pilot trial, so the sample size was modest, although comparable to other reports addressing the learning effects in HF.^12,21,22^ In respiratory medicine, larger cohorts may have enabled more precise estimates of learning effects. In SilHF, baseline tests were not performed on consecutive days; however, the two tests were never more than 2 weeks apart and usually done within a few days. Previous literature suggests that the learning effect may persist for several weeks and gradually diminish over time, potentially being abolished by 6 months.^8^ Therefore, the interval used in SilHF is likely sufficient to capture short-term familiarization. Finally, our cohort comprised symptomatic patients with HFrEF and echocardiographic evidence of PH, and the findings may not apply across the spectrum of patient phenotypes in HF.

### Clinical implications

Establishing whether a single baseline 6MWT is sufficient in HFrEF is important, as it may reduce patient burden and simplify assessment of functional status in clinical trials, while also lowering investigator and staff workload and costs. In clinical practice, this may further support the use of single baseline test when interpreting symptoms-related functional capacity.

## Conclusion

In symptomatic patients with HFrEF and PH, repeat baseline 6MWT testing demonstrated a small but statistically significant increase in walking distance between screening and inclusion. However, the magnitude of improvement was unlikely to be clinically meaningful and did not influence correlations with longitudinal patient-reported outcomes. These findings suggest that, in symptomatic patients with HFrEF, a single baseline 6MWT may be sufficient for assessment of exercise capacity and for interpretation of symptom-related endpoints in both clinical trials and clinical practice.

## Declarations

### Data Availability

No data were generated or analysed for this manuscript.

### Pre-registered Clinical Trial Number

None supplied.

## References

[1] ATS Committee on Proficiency Standards for Clinical Pulmonary Function Laboratories . ATS statement: guidelines for the six-minute walk test. Am J Respir Crit Care Med 2002;166:111–7. 10.1164/ajrccm.166.1.at1102 12091180

[2] Paggiaro PL, Dahle R, Bakran I, Frith L, Hollingworth K, Efthimiou J. Multicentre randomised placebo-controlled trial of inhaled fluticasone propionate in patients with chronic obstructive pulmonary disease. International COPD Study Group. Lancet 1998;351:773–80. 10.1016/s0140-6736(97)03471-5 9519948

[3] Faggiano P, D'Aloia A, Gualeni A, Brentana L, Dei Cas LD. The 6 minute walking test in chronic heart failure: indications, interpretation and limitations from a review of the literature. Eur J Heart Fail 2004;6:687–91. doi:10.1016/j.ejheart.2003.11.024 15542403

[4] Hernandes NA, Wouters EFM, Meijer K, Annegarn J, Pitta F, Spruit MA. Reproducibility of 6-minute walking test in patients with COPD. Eur Respir J 2011;38:261–7. 10.1183/09031936.00142010 21177838

[5] Jenkins S, Cecins NM. Six-minute walk test in pulmonary rehabilitation: do all patients need a practice test? Respirology 2010;15:1192–6. 10.1111/j.1440-1843.2010.01841.x 20920121

[6] Agarwala P, Salzman SH. Six-minute walk test: clinical role, technique, coding, and reimbursement. Chest 2020;157:603–11. 10.1016/j.chest.2019.10.014 PMC7609960 31689414

[7] Holland AE, Spruit MA, Troosters T, Puhan MA, Pepin V, Saey D, et al An official European Respiratory Society/American Thoracic Society technical standard: field walking tests in chronic respiratory disease. Eur Respir J 2014;44:1428–46. 10.1183/09031936.00150314 25359355

[8] Spencer L, Zafiropoulos B, Denniss W, Fowler D, Alison J, Celermajer D. Is there a learning effect when the 6-minute walk test is repeated in people with suspected pulmonary hypertension? Chron Respir Dis 2018;15:339–46. 10.1177/1479972317752762 PMC6234568 29361830

[9] Wu G, Sanderson B, Bittner V. The 6-minute walk test: how important is the learning effect? Am Heart J 2003;146:129–33. 10.1016/S0002-8703(03)00119-4 12851620

[10] Ingle L, Shelton RJ, Rigby AS, Nabb S, Clark AL, Cleland JG. The reproducibility and sensitivity of the 6-min walk test in elderly patients with chronic heart failure. Eur Heart J 2005;26:1742–51. 10.1093/eurheartj/ehi259 15831556

[11] Opasich C, Pinna GD, Mazza A, Febo O, Riccardi PG, Capomolla S, et al Reproducibility of the six-minute walking test in patients with chronic congestive heart failure: practical implications. Am J Cardiol 1998;81:1497–500. 10.1016/S0002-9149(98)00218-5 9645905

[12] Uszko-Lencer N, Mesquita R, Janssen E, Werter C, Brunner-La Rocca HP, Pitta F, et al Reliability, construct validity and determinants of 6-minute walk test performance in patients with chronic heart failure. Int J Cardiol 2017;240:285–90. 10.1016/j.ijcard.2017.02.109 28377186

[13] Cooper TJ, Cleland JGF, Guazzi M, Pellicori P, Ben Gal T, Amir O, et al Effects of sildenafil on symptoms and exercise capacity for heart failure with reduced ejection fraction and pulmonary hypertension (the SilHF study): a randomized placebo-controlled multicentre trial. Eur J Heart Fail 2022;24:1239–48. 10.1002/ejhf.2527 PMC9544113 35596935

[14] Cooper TJ, Anker SD, Comin-Colet J, Filippatos G, Lainscak M, Lüscher TF, et al Relation of longitudinal changes in quality of life assessments to changes in functional capacity in patients with heart failure with and without anemia. Am J Cardiol 2016;117:1482–7. 10.1016/j.amjcard.2016.02.018 27015889

[15] Guyatt GH, Sullivan MJ, Thompson PJ, Fallen EL, Pugsley SO, Taylor DW, et al The 6-minute walk: a new measure of exercise capacity in patients with chronic heart failure. Can Med Assoc J 1985;132:919–23. PMC1345899 3978515

[16] Rabin R, de Charro F. EQ-5D: a measure of health status from the EuroQol Group. Ann Med 2001;33:337–43. 10.3109/07853890109002087 11491192

[17] Calvert MJ, Freemantle N, Cleland JGF. The impact of chronic heart failure on health-related quality of life data acquired in the baseline phase of the CARE-HF study. Eur J Heart Fail 2005;7:243–51. 10.1016/j.ejheart.2005.01.012 15701474

[18] Green CP, Porter CB, Bresnahan DR, Spertus JA. Development and evaluation of the Kansas City Cardiomyopathy Questionnaire: a new health status measure for heart failure. J Am Coll Cardiol 2000;35:1245–55. 10.1016/S0735-1097(00)00531-3 10758967

[19] Jain SS, Cohen DJ, Zhang Z, Uriel N, Sayer G, Lindenfeld JA, et al Defining a clinically important change in 6-minute walk distance in patients with heart failure and mitral valve disease. Circ Heart Fail 2021;14:e007564. 10.1161/CIRCHEARTFAILURE.120.007564 33663234

[20] Shoemaker MJ, Curtis AB, Vangsnes E, Dickinson MG. Clinically meaningful change estimates for the six-minute walk test and daily activity in individuals with chronic heart failure. Cardiopulm Phys Ther J 2013;24:21–9. 10.1097/01823246-201324030-00004 PMC3751711 23997688

[21] Lans C, Cider Å, Nylander E, Brudin L. Test-retest reliability of six-minute walk tests over a one-year period in patients with chronic heart failure. Clin Physiol Funct Imaging 2020;40:284–9. 10.1111/cpf.12637 32337834

[22] Adsett J, Mullins R, Hwang R, Hogden A, Gibson E, Houlihan K, et al Repeat six-minute walk tests in patients with chronic heart failure: are they clinically necessary? Eur J Cardiovasc Prev Rehabil 2011;18:601–6. 10.1177/1741826710389403 21450639

[23] Borgmann M, Ivanda M, Hadizamani Y, Mohaupt M, Bals R, Lucas R, et al Does the 6-minute walk test in hospitalized COPD patients exclusively correlate with lung function parameters or should psychological factors also be taken into account? PLoS One 2020;15:e0232587. 10.1371/journal.pone.0232587 PMC7197854 32365134

[24] Fischer MJ, Scharloo M, Abbink J, van ‘t Hul A, van Ranst D, Rudolphus A, et al Concerns about exercise are related to walk test results in pulmonary rehabilitation for patients with COPD. Int J Behav Med 2012;19:39–47. 10.1007/s12529-010-9130-9 PMC3277820 21080250

[25] Chia KSW, Brown K, Kotlyar E, Wong PKK, Faux SG, Shiner CT. Tired, afraid, breathless….’ An international survey of the exercise experience for people living with pulmonary hypertension. Pulm Circ 2020;10:2045894020968023. 10.1177/2045894020968023 PMC7675876 33240490

